# Lamp2 inhibits epithelial-mesenchymal transition by suppressing Snail expression in HCC

**DOI:** 10.18632/oncotarget.25367

**Published:** 2018-07-13

**Authors:** Hao Zheng, Yuan Yang, Chen Ye, Peng-Peng Li, Zhen-Guang Wang, Hao Xing, Hao Ren, Wei-Ping Zhou

**Affiliations:** ^1^ The Third Department of Hepatic Surgery, Eastern Hepatobiliary Surgery Hospital, Second Military Medical University, Shanghai 200433, China; ^2^ Department of Microbiology, Shanghai Key Laboratory of Medical Biodefense, Second Military Medical University, Shanghai 200433, China; ^3^ Department of Urology, Changhai Hospital, Second Military Medical University, Shanghai 200433, China

**Keywords:** Lamp2, hepatocellular carcinoma, EMT, metastasis

## Abstract

Lysosomal associated membrane protein 2 (Lamp2) influences a broad range of physiological and pathological processes. However, little is known about the role of Lamp2 in hepatocellular carcinoma (HCC) metastasis. This study found that Lamp2 expression was significantly lower in HCC tissues than in adjacent nontumor tissues (ANTs), and its expression level correlated with HCC metastasis. Low Lamp2 expression was significantly correlated with the AFP serum level (> 20 ng/Ml, *P* = 0.024), capsular formation (absent, *P* = 0.024), and microvascular invasion (present, *P* < 0.001), and low expression of Lamp2 indicated a poor prognosis in HCC. LowLamp2 expression was an independent and significant risk factor for recurrence-free survival (RFS; *P* < 0.001) and overall survival (OS; *P* < 0.001) in HCC. In this study, we demonstrated that Lamp2 overexpression inhibited cell motility and invasiveness *in vitro* and inhibited lung metastasis *in vivo*. In addition, Lamp2 could reverse the EMT program. Lamp2 silencing by siRNA in HCC cell lines enhanced the expression of mesenchymal markers and decreased the expression of epithelial markers. Consistent with these findings, Lamp2 overexpression had the opposite effects. Mechanistically, we found that Lamp2 could suppress Snail expression, upregulate E-cadherin, and inhibit HCC cell epithelial-mesenchymal transition (EMT).Together, these findings suggest that Lamp2 attenuates EMT by suppressing Snail expression in HCC.

## INTRODUCTION

Hepatocellular carcinoma (HCC) is one of the commonest malignant tumors and a leading cause of cancer-related death [1, 2]. With improvements in surgical techniques, perioperative management, liver transplantation and adjuvant treatment, the 5-year survival rate for HCC has somewhat increased [3–5]. However, the risks of recurrence and intrahepatic or extrahepatic metastasis after surgery remain high [6]. Invasion and metastasis are the most common causes of death in HCC patients. The pathological mechanism underlying metastasis in HCC remains poorly understood, and there is no effective method for prevention or therapy. Therefore, it is important to understand the mechanisms of HCC metastasis, and novel target markers for early detection and treatment need to be identified.

Increasing evidence suggests that an epithelial-mesenchymal transition (EMT) plays a vital role in metastasis progression in multiple cancers by inducing epithelial cells to adopt mesenchymal attributes [7]. EMT is a complex biological process characterized by the loss of epithelial markers and acquisition of mesenchymal markers that are involved in cancer metastasis [8], including E-cadherin and ZO-1 downregulation as well as N-cadherin and Vimentin upregulation. Moreover, an EMT could be induced by several transcription factors and cytokines, such as Snail, Slug, Twist and TGF-β [9, 10]. Cells undergo substantial changes during the tumor EMT process; for example, epithelial cells lose polarity, and their ability to adhere to the basement membrane decreases while their ability to migrate and invade increases. In addition, the extracellular matrix degrades. As a result, EMT is closely related to tumor invasion and metastasis, but the exact molecular mechanism remains unclear.

Lamp2 is a single-span lysosomal membrane protein that participates in autophagy, maintains lysosomal stability and is critical for lysosomal function [11, 12]. In humans, mutations in theLAMP2 gene cause Danon disease, an X-linked lysosomal storage disorder characterized by the accumulation of vacuolar compartments in heart and skeletal muscle, leading to cardiomyopathy and myopathy [13, 14]. The following three spliced variants of the LAMP2 gene generated by alternative splicing have been described: LAMP-2A, LAMP-2B and LAMP-2C, which differ in the transmembrane and cytoplasmic domains [15]. LAMP-2A functions as a receptor of chaperone-mediated autophagy (CMA), a lysosomal proteolytic process known to be activated during starvation that removes damaged cellular proteins [16]. LAMP-2B is more abundantly expressed in muscle and brain and its absence is associated with Danon disease development. The LAMP-2C isoform functions as a receptor for RNA and DNA degradation [17, 18]. A recent report demonstrated the upregulation of Lamp2 and increased autophagy activity during neuroendocrine differentiation of prostate cancer LNCAP cells [19]. However, the impact of Lamp2 on HCC is poorly understood. Therefore, in this study, we focused on the expression pattern, clinical significance, and function of Lamp2 in HCC.

## RESULTS

### Lamp2 expression is frequently decreased in human HCC tissues

**Figure 1:**
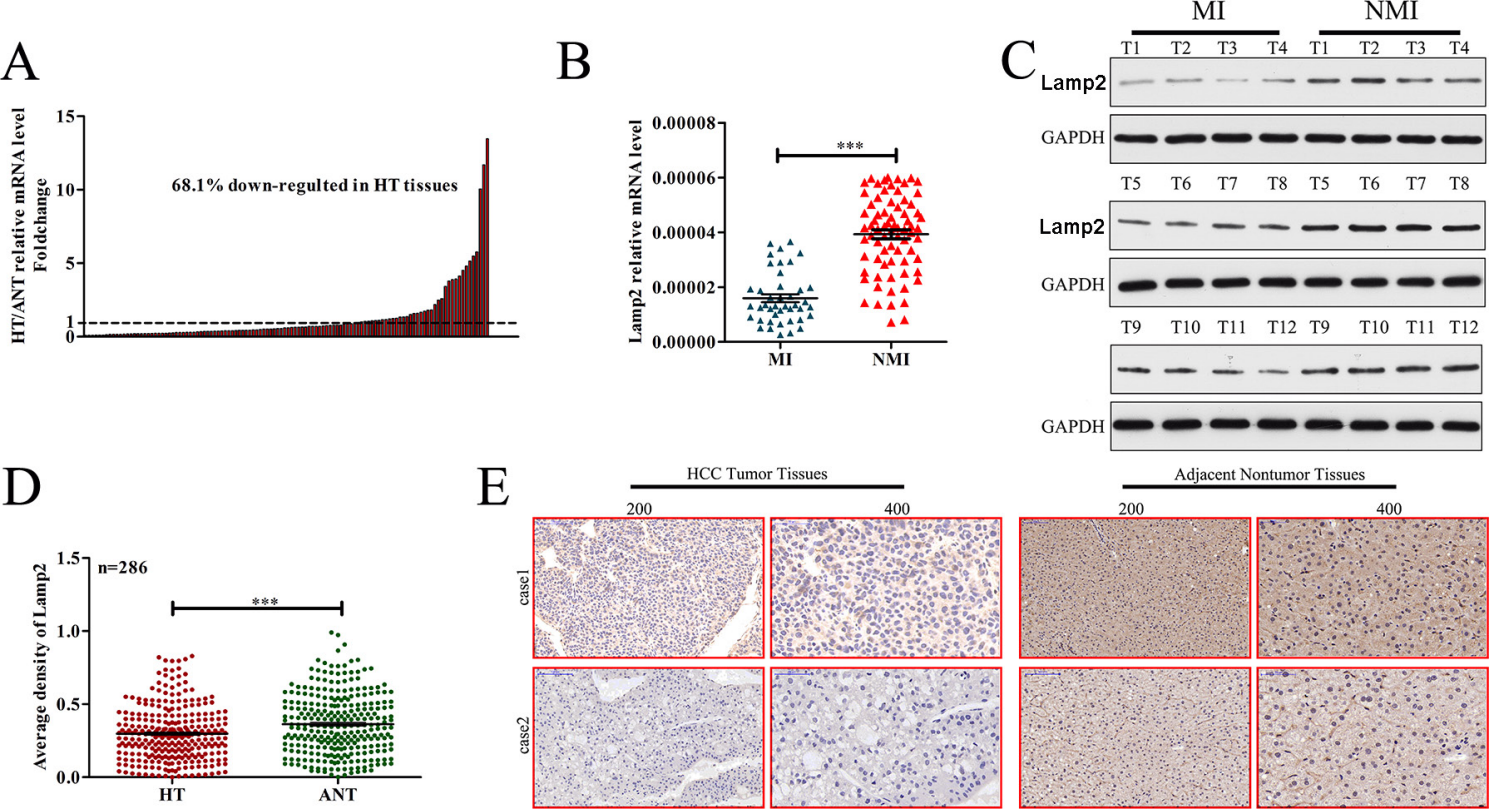
Lamp2 expression is frequently decreased in human HCC tissues (**A** and **B**) Lamp2 mRNA levels in 116 paired HCC and adjacent nontumor tissues were evaluated by qRT-PCR. (**C**) Lamp2 protein levels in MI and NMI HCCs were evaluated by Western blot. (**D**) Relative IHC staining of Lamp2 in paired HCC and adjacent nontumor tissue samples (*n* = 286). (**E**) Two representative cases of Lamp2cIHC staining in HCC and adjacent nontumor tissue pairs in the tissue microarray. (Statistical significance was determined with Student's *t*-tests. ^*^p < 0.05; ^**^p < 0.01; and ^***^p < 0.0001 compared with respective controls. Data are shown as the mean ± SD).

To clarify the significance of Lamp2 expression in HCC, quantitative real-time polymerase chain reaction (qRT-PCR) was used to detect Lamp2 expression in 116 paired fresh HCC samples. The results showed that, compared with matched adjacent nontumor liver tissues (ANLTs), the expression of Lamp2 in HCC tissues (HT) was significantly downregulated (fold change (T/ANLT) > 1) in 68.1% cases (79/116) (Figure 1A). RT-PCR (Figure 1B) and Western blot analysis (Figure 1C) indicated that LAMP2 expression was lower in metastasis-inclined (MI) HCCs than in non-metastasis-inclined (NMI) HCCs. Furthermore, we constructed a tissue microarray containing 286 pairs of HCC and adjacent nontumor tissues for IHC staining. Lamp2 staining was significantly reduced in HCC tissues (Figure 1D and 1E). These data show that Lamp2 is downregulated in HCC and may be associated with HCC metastasis.

### Lamp2 downregulation is negatively associated with malignant clinicopathological characteristics and predicts poorer prognosis in HCC patients following hepatectomy

**Figure 2:**
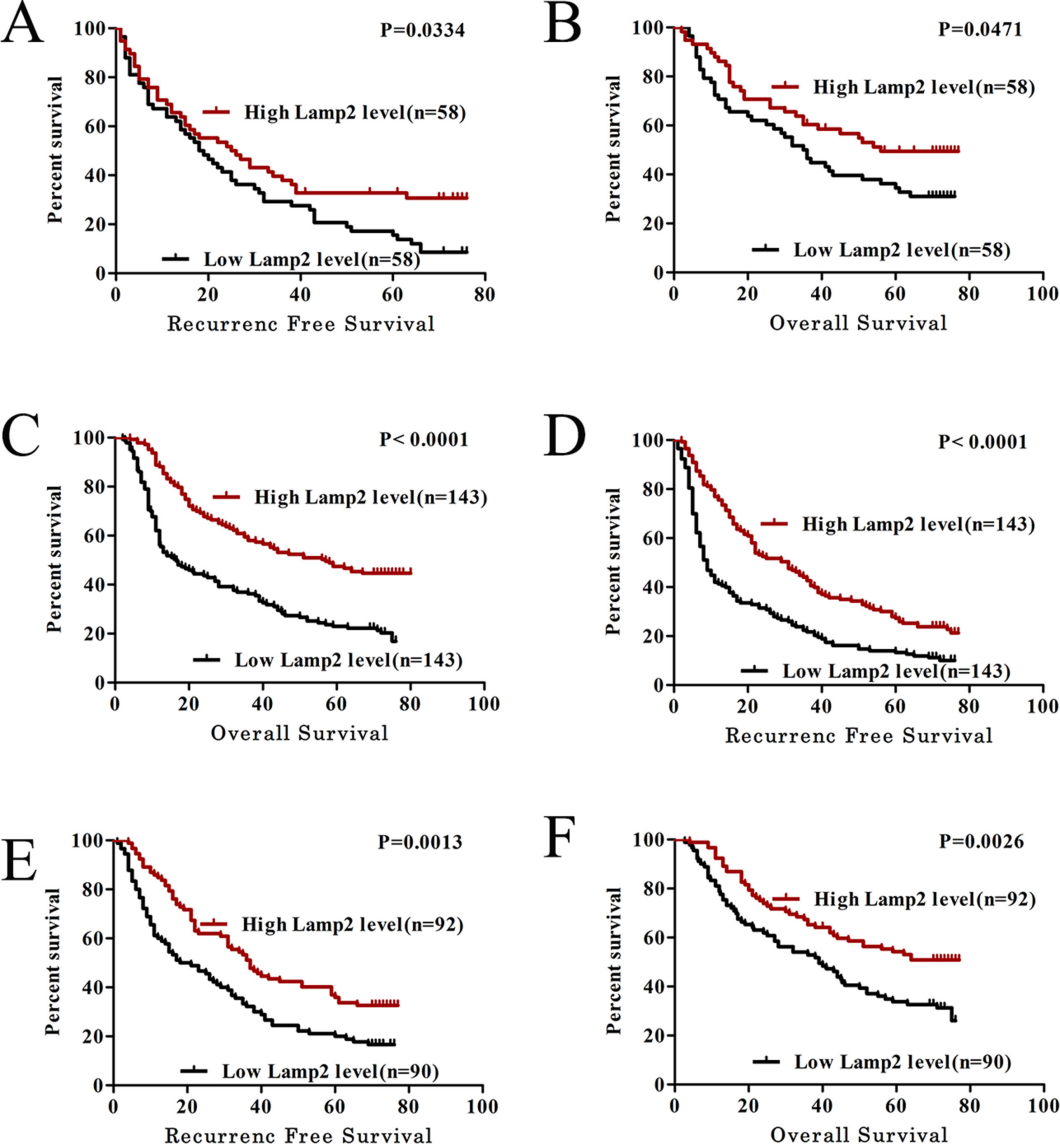
Lamp2 can serve as a prognostic predictor in HCC patients (**A** and **B**) The low Lamp2 subgroup (*n* = 58) had a significantly shorter RFS and OS than the high Lamp2 subgroup (*n* = 58) in the qRT-PCR cohort. (**C** and **D**) Similar results were observed in the tissue microarray cohort: the low Lamp2 subgroup (*n* = 143) vs the high Lamp2 subgroup (*n* = 143). (**E** and **F**) The prognostic value of LAMP2 was also observed in patients with early-stage HCC (TNM stage I): the low Lamp2 subgroup (*n* = 90) vs. the high Lamp2 subgroup (*n* = 92). Statistical significance was assessed by two-sided log-rank tests.

To further investigate the clinical significance of Lamp2 expression in the development and progression of HCC, the tissue microarray cohort (286 patients) was divided into 2 groups based on the overall expression level of Lamp2: a high Lamp2 expression group (*n* = 143) and a low Lamp2 expression group (*n* = 143).We found strong negative correlations between Lamp2 expression and many progressive clinical features, including the AFP serum level (> 20 ng/Ml, *P* = 0.024), capsular formation (absent, *P* = 0.024), and microvascular invasion (present, *P* < 0.001) (Table 1). To determine the prognostic value of Lamp2 in HCC, Kaplan-Meier survival curves were generated and log-rank tests were performed in both qRT-PCR and tissue microarray cohorts. The median expression level was used as the cutoff. Patients with lower Lamp2 mRNA levels had a significantly shorter RFS (*P* = 0.0334) and OS (*P* = 0.0471) than patients with higher Lamp2 mRNA levels (Figure 2A and 2B). Moreover, there were similar findings in the tissue microarray cohort (*P* < 0.0001 for RFS, *P* < 0.0001 for OS) (Figure 2C and 2D). A univariate analysis indicated that among the clinicopathological characteristics, age, the Lamp2 expression level, AFP serum level, tumor number, microvascular invasion, and tumor diameter were correlated with RFS, and the Lamp2 expression level, liver cirrhosis, Edmondson-Steiner classification, microvascular invasion, tumor number, tumor diameter, and AFP serum level were correlated with the OS (Supplementary Table 1). Furthermore, multivariate Cox regression analysis indicated that the Lamp2 expression level, tumor diameter, tumor number, and microvascular invasion were correlated with both the RFS and OS (Table 2). The prognosis of some patients with early-stage HCC has been poor, suggesting that a supplementary prognostic predictor is required for these patients. Therefore, patients with early-stage HCC (TNM stage I) were stratified, and subgroup analyses were performed. Notably, the prognosis-predictive value of Lamp2 in early-stage HCC (TNM stage I) was demonstrated (*p* = 0.0013 for RFS and *p* = 0.0026 for OS) (Figure 2E and 2F). Similar results were also observed in patients with a normal serum AFP level (< 20 μg/L) and small hepatocellular carcinoma (SHCC, the diameter of HCC ≤ 5 cm) (Supplementary Figure 1A–1D). Taken together, these data indicate that the expression level of Lamp2 can be used as an independent factor for predicting the prognosis of HCC.

**Table 1:** Correlation of the Lamp2 protein level in HCC tissues with clinicopathological characteristics

Factors	Lamp2 expression		χ^2^	*P*-value
	High	Low		
**Gender**				
Male	132	126	1.425	0.233
Female	11	17		
**Age (years)**				
> 55	60	64	0.228	0.633
≤ 55	83	79		
**AFP (ng/Ml)**				
> 20	51	84	15.279	< 0.001^#^
≤ 20	92	59		
**HBsAg**				
Positive	125	122	0.267	0.605
Negative	18	21		
**Liver cirrhosis**				
Present	87	92	0.373	0.541
Absent	56	51		
**Tumor number**				
Multiple	43	45	0.066	0.798
Solitary	100	98		
**Tumor size (cm)**				
> 5	82	84	0.057	0.811
≤ 5	61	59		
**Capsular formation**				
Present	89	70	5.113	0.024^#^
Absent	54	73		
**Microvascular invasion**				
Present	61	93	14.407	< 0.001^#^
Absent	82	50		
**Edmondson-Steiner grade**				
Low grade (I and II)	22	15	1.521	0.217
High grade (III and IV)	121	128		
**TNM Stage**				
I	92	90	0.06	0.806
II–III	51	53		
**BCLC Stage**				
A+B	77	80	0.127	0.721
C+D	66	63		

Values shown with ^#^ are statistically significant (*p* < 0.05).

The median expression level was used as the cutoff value. Low Lamp2 expression in each of the 143 patients was defined as a value below the 50th percentile. High Lamp2 expression in each of the 143 patients was defined as a value above the 50th percentile.

Pearson's Chi-square tests were used for the correlation analysis between the expression levels of Lamp2 and clinical features. The results were considered statistically significant at *p* < 0.05.

**Table 2:** Multivariate analysis of the risk factors for RFS and OS in 286 HCC patients

Variable	RFS				OS			
	*P*	HR	95% CI		*P*	HR	95% CI	
**Tumor diameter**, > 5 cm vs. ≤ 5 cm	< 0.001	2.372	1.771	3.177	< 0.001	1.540	1.113	2.130
**Tumor number**, Multiple vs. Solitary	< 0.001	2.580	1.914	3.478	< 0.001	2.632	1.889	3.667
**Microvascular invasion**, present vs. absent	< 0.001	1.830	1.394	2.402	< 0.001	2.968	2.185	4.032
**Lamp2 expression**, low vs. high	< 0.001	2.786	2.103	3.692	< 0.001	3.733	2.695	5.171

### Lamp2 inhibits HCC cell migration and invasion

**Figure 3:**
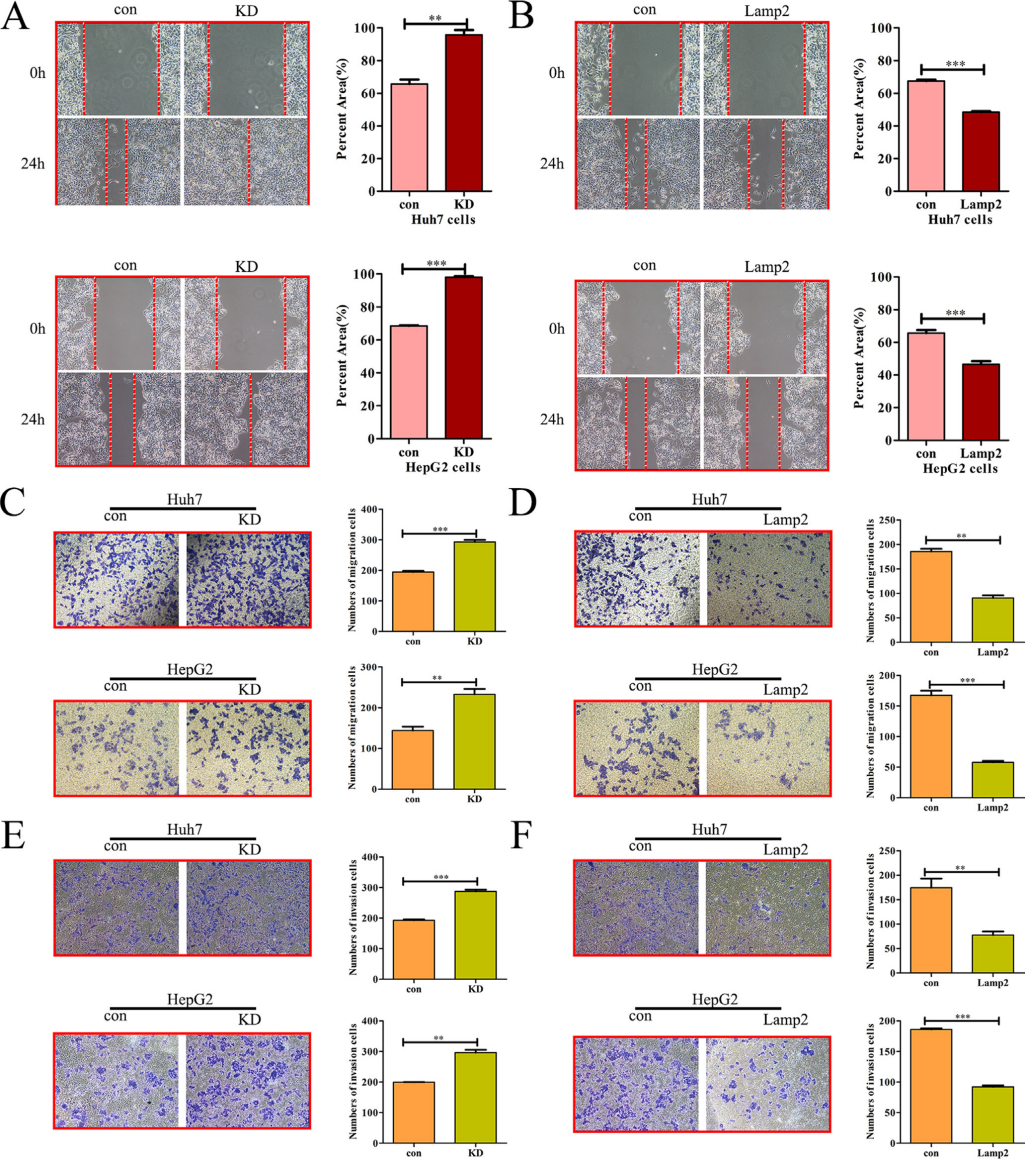
Lamp2 inhibits the migration and invasion of HCC cells (**A**, **C** and **E**) Wound-healing, Transwell migration assays and Matrigel invasion assays in Lamp2-silenced Huh7 and HepG2 cells. (**B**, **D**, **F**) Cell migration and Invasion assays in Huh7 and HepG2 cells overexpressing Lamp2. Cells were counted in 3 randomized fields at a magnification of 100×. The error bar represents the mean ± SD of triplicate assays (^*^p < 0.05, ^**^p < 0.01, and ^***^p < 0.001; *p*-values were calculated using Student's *t*-test).

To explore the effects of Lamp2 on the invasiveness of HCC cells, we examined the expression of Lamp2 in six HCC cell lines (SMMC-7721, MHCC97L, Huh7, HepG2, HCCLM3 and MHCC97H) that exhibit different invasive behaviors. The Lamp2 mRNA and protein levels were highest in SMMC-7721 and MHCC97L cells (low metastatic potential), decreased in Huh7 and HepG2 cells, and lowest in HCCLM3 and MHCC97H cells (high metastatic potential) (Supplementary Figure 2A and 2B). To further evaluate the role of Lamp2 in HCC cell migration and invasion, we established stable cell lines (Huh7 and HepG2 cells) that were transfected with siRNA-NC (referred to as Huh7-Con and HepG2-Con) or siRNA-Lamp2 (referred to as Huh7-KD and HepG2-KD). In addition, theHuh7 and HepG2 cell lines were infected with the LV-NC lentivirus (referred to as Huh7-Con and HepG2-Con) or LV-Lamp2 lentivirus (referred to as Huh7-Lamp2 and HepG2-Lamp2). RT-PCR and Western blot analysis indicated that Lamp2 expression was silenced by siRNA-Lamp2 and upregulated by Lamp2 overexpression (Supplementary Figure 2C–2E). Lamp2 silencing increased the wound-healing capability of Huh7 and HepG2 cells, while Lamp2 overexpression reduced the wound-healing capability of Huh7 and HepG2 cells (Figure 3A and 3B). Consistent with these findings, a Transwell assay demonstrated that Lamp2 silencing significantly enhanced the migration and invasion of Huh7 and HepG2 cells (Figure 3C and 3E). Conversely, Lamp2 overexpression markedly inhibited the migration and invasion of Huh7 and HepG2cells (Figure 3D and 3F). Together, these data indicate that Lamp2 inhibits the motility and invasiveness of HCC cells.

### Lamp2 overexpression in luciferase-labeled Huh7 and HepG2 cells inhibits lung metastasis

**Figure 4:**
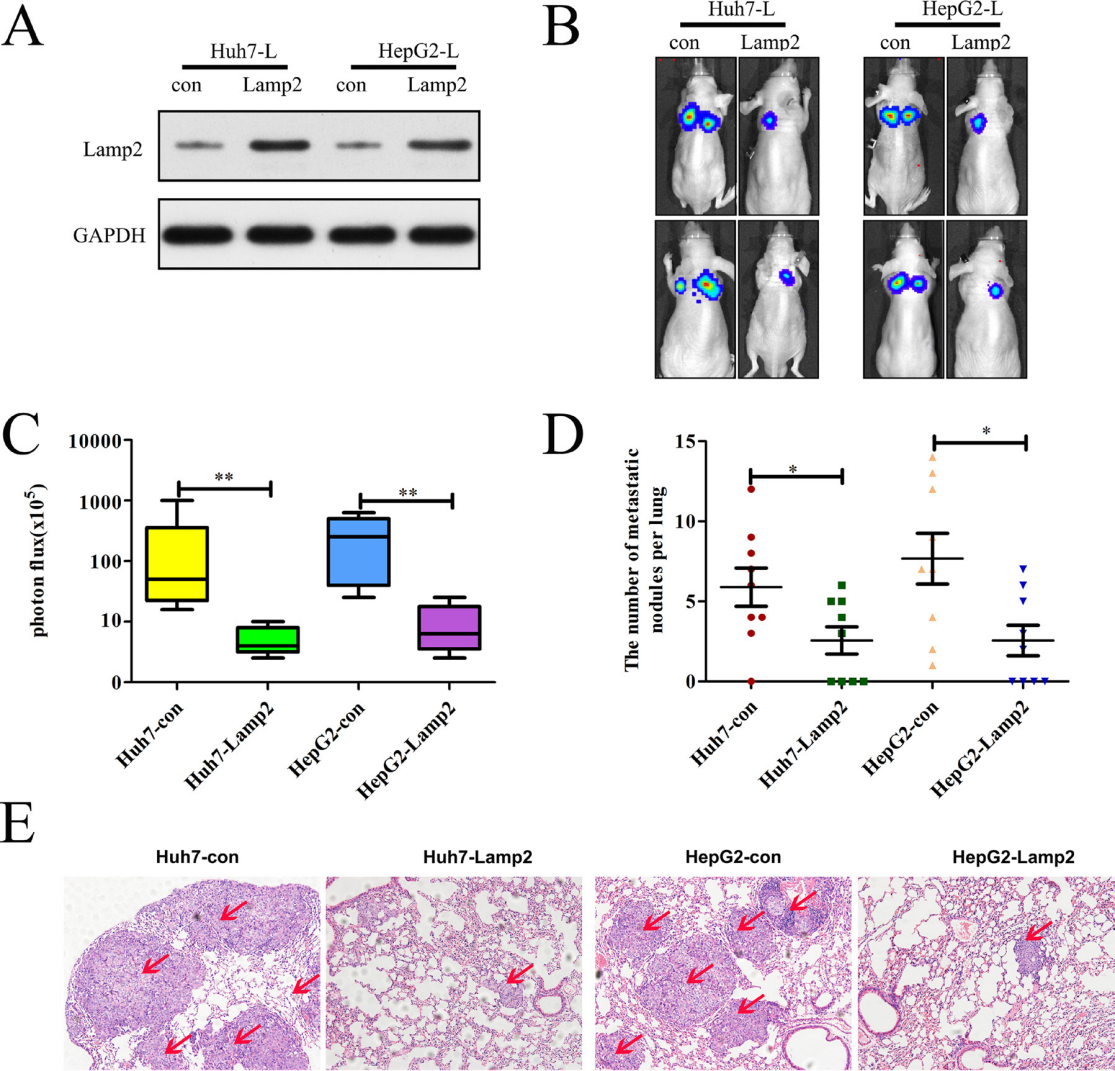
Lamp2 overexpression in luciferase-labeled Huh7 and HepG2 cells inhibits lung metastasis (**A**)Western blot analysis of Lamp2 expression in luciferase-labeled Huh7 and HepG2 cells that were infected with empty vector or LV-Lamp2. (**B**)The 8 nude mice in each group were imaged with bioluminescence 6 weeks after the injection of HCC cells, and representative images for each group are shown. (**C**) The statistical analysis of bioluminescence in mice. (**D**) The number of lung metastatic foci in each group. (**E**) Representative images of H&E-stained lung tissue samples from each group. Scale bar indicates 200 μm. (^*^p < 0.05, ^**^p < 0.01, and ^***^p < 0.001; *p*-values were calculated using Student's *t*-test).

To investigate the role of Lamp2 *in vivo*, we injected stably transfected cell lines (Huh7-Con, HepG2-Con, Huh7-Lamp2 and HepG2-Lamp2) into the tail vein of nude mice and evaluated the presence of lung metastatic nodules. The expression of Lamp2 was verified by Western blotting, which showed that Lamp2 expression in Huh7-Lamp2and HepG2-Lamp2 cells was increased compared with the control (Figure 4A). Bioluminescent imaging of nude mice demonstrated the incidence of lung metastasis was lower in mice injected with Huh7-Lamp2 or HepG2-Lamp2 cells compared with mice injected with Huh7-Con or HepG2-Con cells (Figure 4B and 4C). Moreover, mice injected with Huh7-Lamp2 or HepG2-Lamp2 cells had fewer and smaller lung metastases compared to the control group (Figure 4D). Histologic analyses of the dissected lungs further confirmed the presence of metastases (Figure 4E). These results demonstrate that Lamp2 plays a critical role in inhibiting HCC invasion and metastasis.

### Lamp2 represses the epithelial-mesenchymal transition in HCC cells

**Figure 5:**
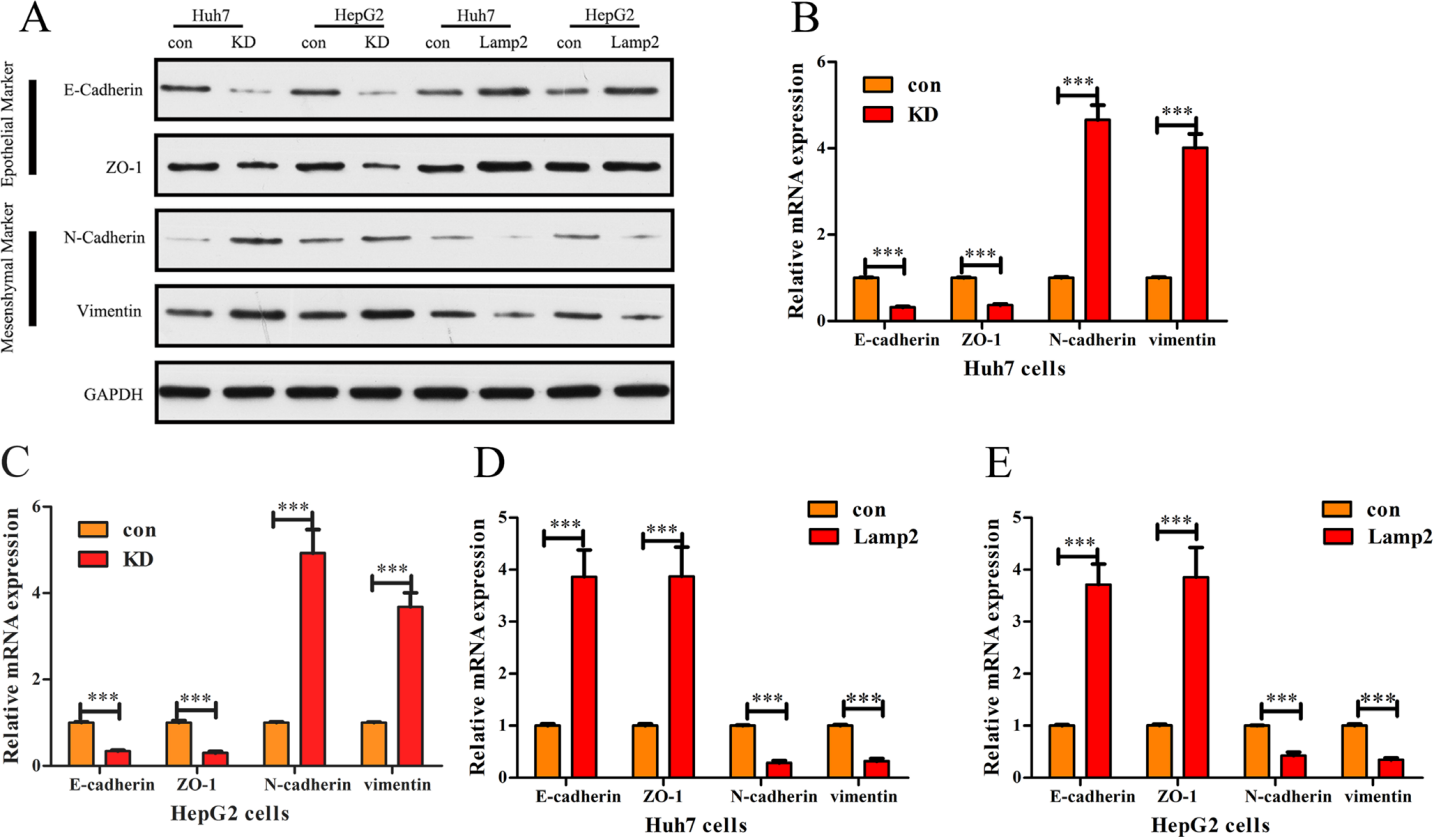
Lamp2 represses the epithelial-mesenchymal transition in HCC cells (**A**, **B** and **C**) Western blot analysis and RT-PCR reveal downregulation of the expression of epithelial markers (E-cadherin and ZO-1) and upregulation of the expression of mesenchymal markers (Vimentin and N-cadherin) in Huh7 andHepG2 cells transfected with Lamp2 siRNA. In contrast, (A) Western blot analysis and (**D**–**E**) RT-PCR reveal that Lamp2 overexpression upregulated the expression of epithelial markers and decreased the expression of mesenchymal markers in Huh7 andHepG2 cells. Scale bar indicates 25 μm.

Given that upregulated Lamp2 correlated with inhibited the cell migratory and invasiveness abilities of HCC cells, we examined the EMT as an underlying mechanism. In Huh7 and HepG2 cells, Lamp2 shRNA downregulated epithelial markers E-cadherin and ZO-1 and upregulated mesenchymal markers N-cadherin and Vimentin. In contrast, overexpression of Lamp2 upregulated epithelial markers E-cadherin and ZO-1 and downregulated mesenchymal markers N-cadherin and Vimentin (Figure 5A). We also detected EMT marker expression using qRT-PCR and found that Lamp2 shRNA downregulated epithelial markers E-cadherin and ZO-1 and upregulated mesenchymal markers N-cadherin and Vimentin (Figure 5B and 5C), whereas overexpression of Lamp2 upregulated epithelial markers E-cadherin and ZO-1 and downregulated mesenchymal markers N-cadherin and Vimentin (Figure 5D and 5E) in Huh7 and HepG2 cells.

### Lamp2 inhibits TGF-β-induced epithelial-mesenchymal transition

**Figure 6:**
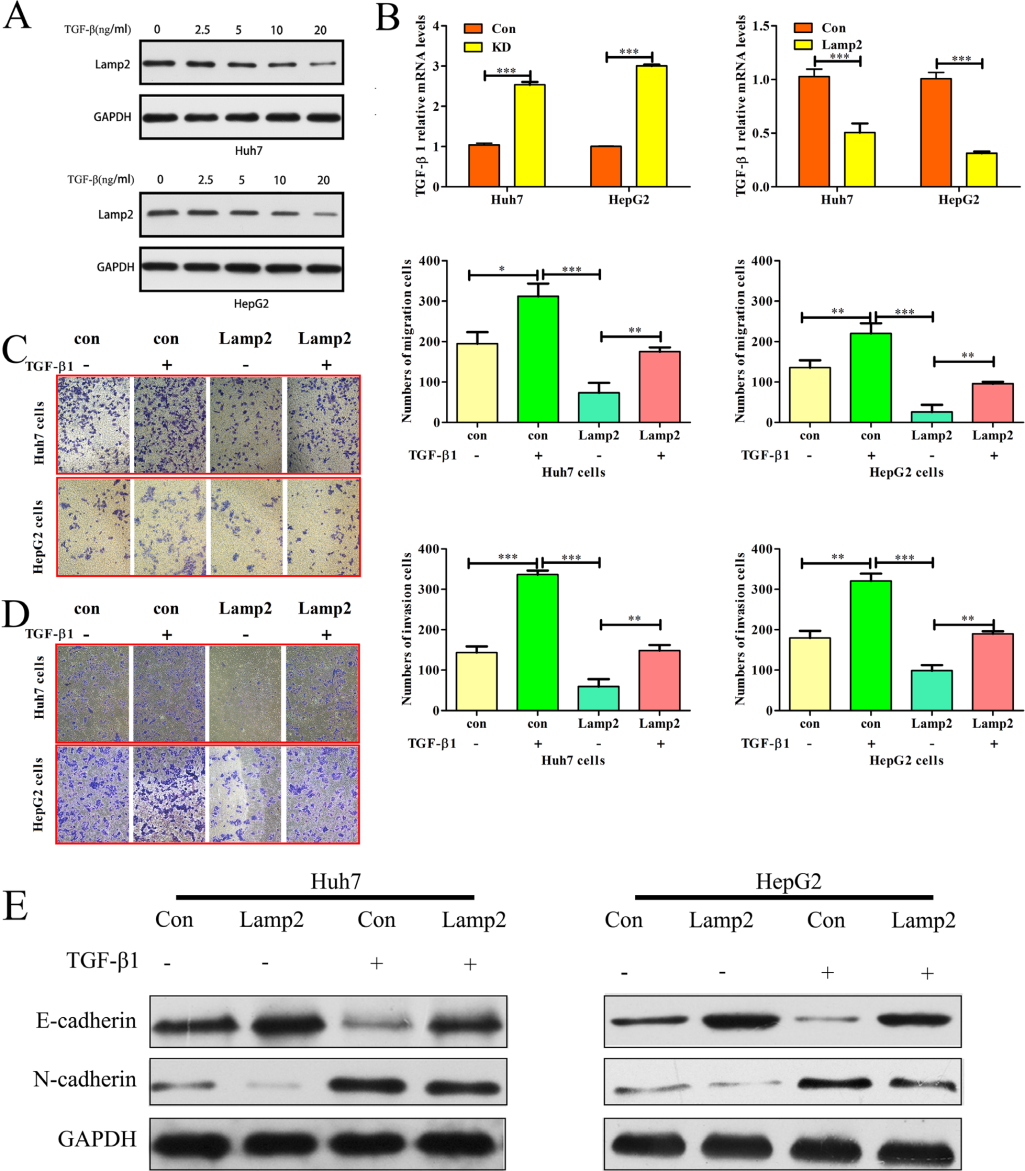
Lamp2 inhibits TGF-β-induced epithelial-mesenchymal transition (**A**) Lamp2 expression levels in Huh7 and HepG2 cells treated with different TGF-β1doses. (**B**) TGF-β1 mRNA levels were determined using real-time PCR in HCC cells treated with si-NC, siRNA-Lamp2, empty vector or LV-Lamp2. The error bar represents the mean ± SD of triplicate assays. (^*^p < 0.05, ^**^p < 0.01, and ^***^p < 0.001; *p*-values were calculated using Student's *t*-test). Relative changes in the number of Huh7 and HepG2 cells that migrated (**C**) and invaded (D) after TGF-β1 treatment or Lamp2 overexpression. (^*^p < 0.05, ^**^p < 0.01, and ^***^p < 0.001; *p*-values were calculated using Student's *t*-test). (**E**). Western blot analysis of Lamp2, E-cadherin and N-cadherin expression after TGF-β1 treatment (20 ng/ml) in Huh7 and HepG2 cells that were infected with the LV-NC or LV-Lamp2 lentivirus.

The TGF-β signaling pathways are associated with the development of liver cancer [20]. We next investigated whether TGF-β signaling-induced EMT was repressed by Lamp2. Western blot analysis was used to determine the level of Lamp2 protein in response to different doses of TGF-β1 in Huh7 and HepG2 cells. Lamp2 expression decreased in response to low doses of TGF-β1 and was lowest for 20 ng/ml TGF-β1 (Figure 6A). Real-time PCR revealed that TGF-β1 expression was increased in Lamp2-silenced HCC cell lines. In contrast, TGF-β1 expression was decreased in HCC cell lines overexpressing Lamp2 (Figure 6B). These data indicate that TGF-β1 and Lamp2 can repress one another. Moreover, HCC cells treated with TGF-β1 had increased cell migration and invasion, which was greatly attenuated by Lamp2overexpression (Figure 6C and 6D). Lamp2 overexpression enhanced E-cadherin expression, which persisted even after TGF-β1 treatment. In addition, N-cadherin expression was inhibited in the Lamp2-overexpressing cells. The Lamp2-induced decrease in N-cadherin expression was inhibited by TGF-β1treatment (Figure 6E). These results suggest that the effects of Lamp2 associated with TGF-β1 more potently affect E-cadherin expression compared to N-cadherin expression and indicate that Lamp2 expression in HCC cells inhibits TGF-β signaling.

### Lamp2 reduced the development of the EMT via suppressing Snail expression

**Figure 7:**
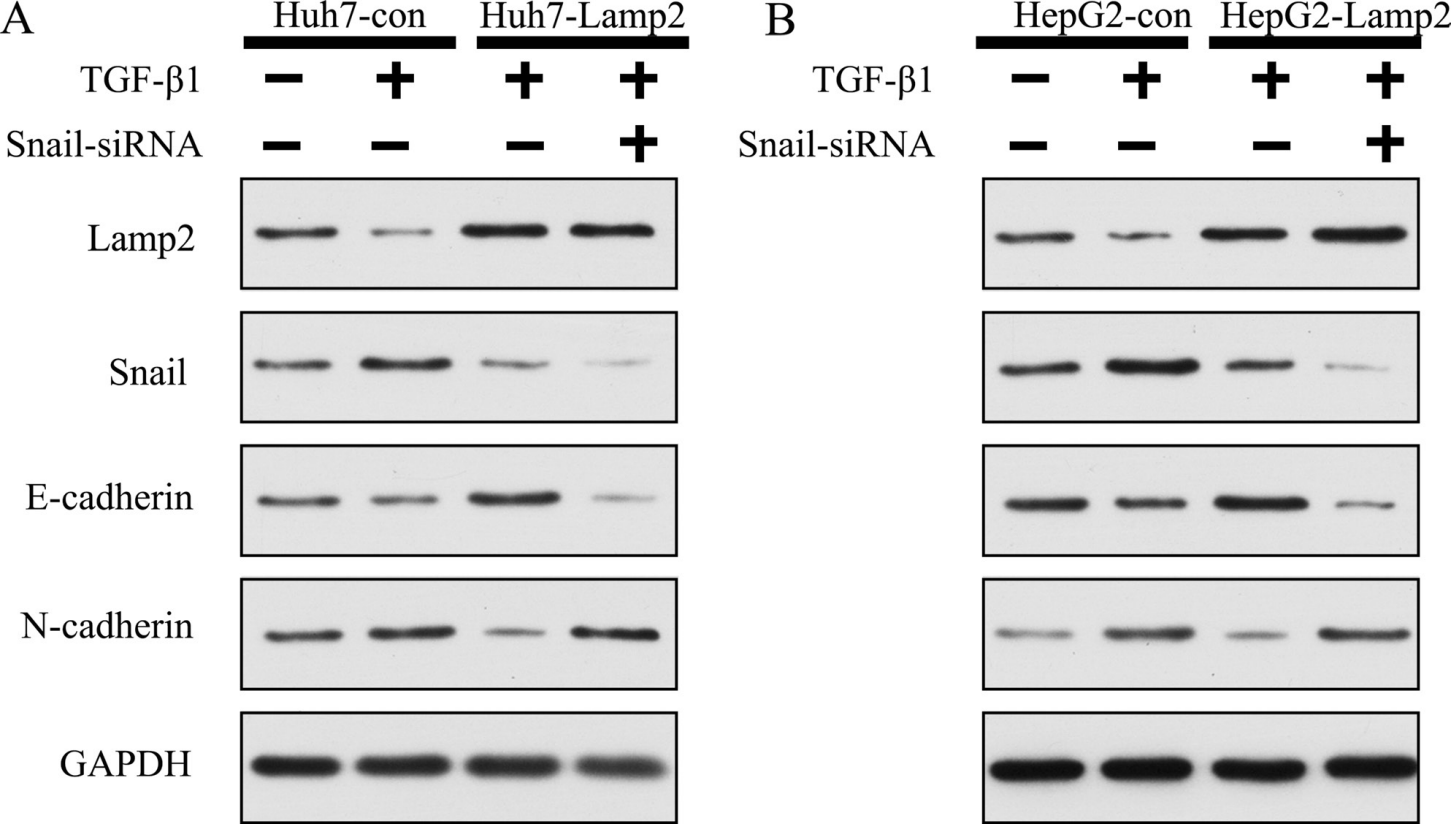
Lamp2 reduced the development of EMT via suppressing Snail expression (**A** and **B**) Snail siRNA was transfected in Huh7 and HepG2 cells for 24 h, which was followed by stimulation with TGF-β 1 for 24 h. Cell lysates were collected, and the relative protein levels were determined by Western blot analysis.

Because Snail is the key inhibitor of E-cadherin transcription [21], we next investigated whether Lamp2 regulated Snail-induced inhibition of E-cadherin transcription. Huh7-Lamp2 and HepG2-Lamp2 cells were transfected with Snail siRNA for 24 h, stimulation with TGF-β1 for 24 h. We found that when Snail was silenced by siRNA, Huh7-Lamp2 and HepG2-Lamp2 cells were unable to reduce the progression of EMT, which was reflected by the upregulation of N-cadherin and downregulation of E-cadherin (Figure 7A and 7B). These findings demonstrate that Lamp2 regulates EMT progression by suppressing Snail expression.

## DISCUSSION

Metastasis is the leading cause of mortality in HCC and a key cause of poor postoperative prognosis in patients with HCC [22–24]. Understanding the mechanisms of HCC metastasis will improve therapeutic strategies for HCC and the postoperative prognosis of HCC patients [25]. Lamp2 influences a broad range of physiological and pathological processes [26, 27]. However, no studies have reported the roles of Lamp2 in HCC progression. To the best of our knowledge, this is the first study to illustrate the roles of Lamp2 in HCC metastasis. In this study, we found that Lamp2 expression was frequently downregulated in HCC, and there was a negative correlation between Lamp2 expression and advanced clinicopathological features. In addition, Lamp2 expression correlated with poor overall and recurrence-free survival in patients diagnosed with HCC. Additionally, we demonstrated that Lamp2 plays a key role in repressing HCC invasion and metastasis. Furthermore, the development of tumor lung metastases was suppressed *in vivo* in nude mice injected with human HCC cells. By overexpressing or silencing Lamp2 in HCC cells, we confirmed that Lamp2 can regulate the expression of EMT markers and transcriptional activators. We also demonstrated that Lamp2 primarily inhibits EMT in HCC by downregulating Snail.

EMT is a vital step in the initiation of metastasis [28–31], and TGF-β1 promotes cell migration, invasion, and metastasis by inducing an EMT [32]. In this study, we found that TGF-β1 suppressed Lamp2 expression in HCC cells and that Lamp2 overexpression decreased the TGF-β expression. Lamp2 can inhibit the process of TGF-β-induced EMT. Therefore, we speculated that a Lamp2-TGFβ feedback loop might represent a novel signaling pathway that regulates EMT and HCC progression. Previous studies have reported that EMT can be mediated by activating a series of transcriptional regulators, such as Snail, Slug, and Twist, and these transcription factors have been found to play crucial roles in promoting EMT [33]. Snail is the most extensively studied, and the Snail protein is one of most important transcription factors to repress CDH1 gene (encoding E-cadherin protein) transcription and induce EMT [34]. Increasing evidence shows that inducing the expression of Snail promotes epithelial-mesenchymal transition and cell invasion [35]. Here, we explored the relationship between Lamp2 and Snail in HCC cells. We proposed that Lamp2 interferes with the process of EMT by inhibiting Snail expression. Nevertheless, the precise mechanisms by which Lamp2 regulates Snail expression require further exploration.

In summary, our study shows that Lamp2 is significantly downregulated in HCC and low Lamp2 expression is associated with a poor prognosis and an EMT phenotype in HCC tissues and cell lines. We observed that Lamp2 protects against the development of EMT by suppressing Snail expression in HCC. Therefore, Lamp2 is a potential therapeutic target for preventing or attenuating EMT in HCC.

## MATERIALS AND METHODS

### Patient characteristics and tissue specimens

All patients in our study had HBV infection. We obtained 116 paired samples of HCC and adjacent nontumor tissues for qRT-PCR analysis. A tissue microarray containing 286 pairs of HCC and adjacent nontumor tissues was constructed for the IHC test. HCC differentiation was defined according to the Edmondson-Steiner classification. Micrometastases were defined as tumors adjacent to the border of the primary tumor, which could only be observed under a microscope. Tumor staging was conducted according to the BCLC staging system and the sixth edition of the Tumor-Node-Metastasis (TNM) classification system published by the International Union Against Cancer. All tissue samples were randomly collected at the Eastern Hepatobiliary Surgery Hospital (Shanghai, China) from September 2005 to March 2012 [36] and were stored at −80°C until further use. Two pathologists independently re-evaluated the tissues. The study was approved by the ethics committee of the Eastern Hepatobiliary Surgery Hospital, and written informed consent was obtained from all study participants according to the committee policies. The RFS was calculated from the date of tumor resection until the detection of tumor recurrence, death from a non-HCC cause, or final follow-up visit. The OS was defined as the length of time between surgery and either patient death or the final follow-up visit. Information that could reveal the identity of the patients was excluded from this report.

### Cell culture

The hepatocellular carcinoma cell lines (SMMC-7721, HCC-LM3, Huh7, HepG2, MHCC97L, and MHCC97L) were obtained from the China Center for Type Culture Collection (Wuhan, China) and were authenticated by the provider using DNA fingerprinting or isoenzyme analysis. All cell lines were maintained in DMEM medium (HyClone, UT, USA) containing 10% fetal bovine serum and 1% penicillin/streptomycin (Gibco BRL, MD, USA) at 37°C in a humidified atmosphere with 5% CO_2_.

### RNA extraction, preparation of cDNA, and qRT-PCR analysis

Total RNA was extracted from frozen tissues or cell lines using Trizol reagent (Takara, Dalian, China) according to the manufacturer's instructions. For reverse transcriptions, 1–2 μg of total RNA, random primers, and the M-MLV Reverse Transcriptase Kit (Invitrogen, CA, USA) were used. Real-time PCR was performed using the SYBR Green Master Mix (Takara, China) in a StepOnePlus system (Applied Biosystems, CA, USA) with β-actin as an endogenous control. The qRT-PCR primers are listed in Supplementary Table 2.

### Western blotting

Western blot analysis was performed as described previously [37]. The cells were harvested and lysed in RIPA buffer. Equal amounts of protein were separated on a sodiumdodecyl sulfate-polyacrylamide gel electrophoresis (SDSPAGE) gel and then transferred to a PVDF membrane. The membrane was incubated in blocking solution (5% milk/TBST) and then incubated with the primary antibody overnight. The membrane was washed 3 times for 10 min with TBST and subsequently incubated with the HRP-conjugated secondary antibody for 1 or 2 h. Then, the membrane was washed 3 times for 10 min with TBST. Changes in protein expression were determined using the Chemiluminescent Substrate (Thermo Scientific).The antibodies against Lamp2, E-cadherin, ZO-1, Vimentin, N-cadherin, ZO-1, Snail and GAPDH used for Western blotting were obtained from (Abcam, MA, USA). The antibody against Lamp2that was used for chromatin immunoprecipitation was obtained from Abcam (MA, USA).

### Tissue microarray, immunohistochemistry and immunohistochemical quantification

Tissue microarrays (TMAs) containing 286 pairs of HCC and adjacent nontumor tissues were constructed in collaboration with the Shanghai Outdo Biotech Company (Shanghai, China). Immunohistochemistry was performed as previously described [38]. A Lamp2 polyclonal antibody (Abcam, MA, USA) diluted at 1:300 was used as the primary antibody. The density of Lamp2-positive staining was quantified using a DFC420 CCD camera connected to a DM IRE2 microscope (Leica Microsystems Imaging Solutions Ltd, Cambridge, United Kingdom). Photographs of representative fields were captured at high-power magnification (200×, 400×) using Leica QWin Plus v3 software. Image-Pro Plus v6.0 software (Media Cybernetics Inc, MD, USA) was used to count the integrated optical density (IOD) of each photograph as previously described [39], and the ratio of IOD to total tissue area (AREA) of each photograph was calculated as the Lamp2 density.

### Transwell assays

Invasion and migration assays were conducted using Transwell chambers (BD Biosciences, SanJose, CA, USA). Cells were suspended in serum-free medium and seeded into the upper compartment of the chamber, while the lower compartment was filled with complete medium. The chamber was incubated for 24–72 h at 37°C with 5% carbon dioxide (CO_2_). For the invasion assay, polycarbonate membranes (8-μm pore size) in the upper compartment of 24-well Transwell culture chambers were coated with 5 mg/ml Matrigel (BD Biosciences). At the end of the incubation, the cells on the upper surface of the filter were removed by scrubbing. The invasive and migratory cells were stained with 0.5% crystal violet for 5 minutes and counted under a light microscope. Each assay was performed in triplicate.

### Wound-healing assay

Cell mobility was evaluated using a wound-healing assay. HCC cells were grown to full confluence in 6-wellplates, and a wound was created by scratching the length of the well with a 10-μl pipette tip. Cells were treated with Mitomycin (20 μg/ml) for 20 min. Then, the cells were washed 3 times with PBS and subsequently incubated with complete medium. Images were captured using an inverted digital camera at 0 h, 24 h and 48 h after the wound was generated. Using ImageJ software, cell migration was quantified by measuring the number of cells that migrated into the wound area at each time point.

### Animal studies

A tail vein injection model was also used to evaluate the potential of the cells to metastasize to the lungs. The metastases were monitored using an IVIS@ Lumina II system (CaliperLife Sciences, Hopkinton, MA) for 10 min after intraperitoneal injection of 4.0 mg of Luciferin (Gold Biotech) in 50 μl of saline. Animals were housed in cages under standard conditions, and the methods were performed according to the requirements of the Second Military Medical University Animal Care Facility and the National Institutes of Health guidelines. All experimental protocols were approved by the Institutional Animal Care and Use Committee of the Second MilitaryMedical University, Shanghai, China. The mice were kept in pathogen-free conditions.

### Statistical analysis

All statistical analyses were performed using SPSS version 17.0 and GraphPad Prism 5.0 software. For qualitative variables, the χ^2^ test or Fisher exact test was used. For continuous variables, Student's *t*-test or the Mann-Whitney test was performed as appropriate. Survival curves were calculated according to the Kaplan-Meier method and were compared by a log-rank test. The Cox's proportional hazards model was used to determine independent factors for survival and recurrence based on variables selected after the univariate analysis. Two-tailed tests were performed to generate *p*-values, and *p* < 0.05 was considered statistically significant.

## SUPPLEMENTARY MATERIALS FIGURES AND TABLES


