# Supplementary material for: Lamp2 inhibits epithelial-mesenchymal transition by suppressing Snail expression in HCC
Source: Oncotarget. 2018 Jul 13;9(54):30240–52. doi: 10.18632/oncotarget.25367 (PMC6084387; doi:10.18632/oncotarget.25367)
Supplement: Supplementary file 1 [file oncotarget-09-30240-s001.pdf]

Lamp2 inhibits epithelial-mesenchymal transition by suppressing Snail expression in HCC

SUPPLEMENTARY MATERIALS

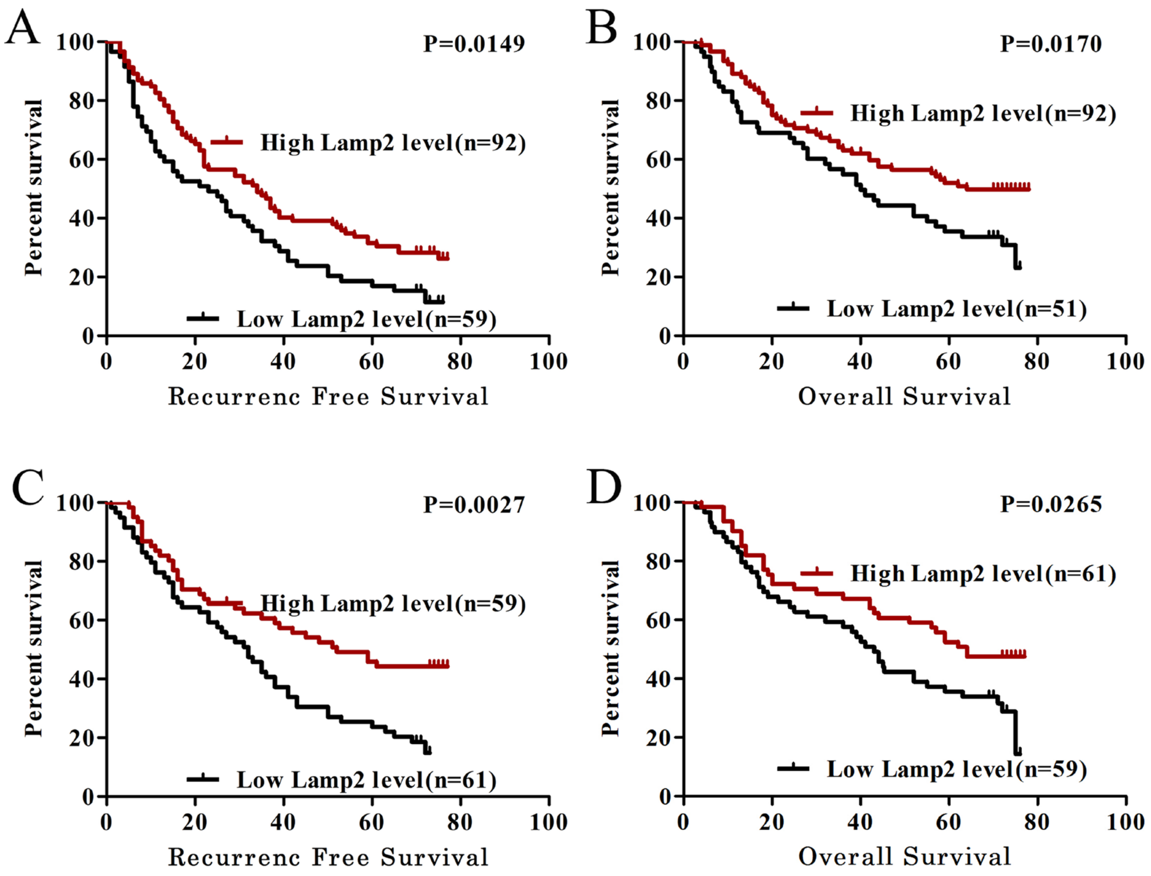

Supplementary Figure 1: (**A** and **B**) The low Lamp2 subgroup had a significantly shorter RFS and OS than the high Lamp2 subgroup in the tissue microarray cohort with a normal serum AFP level (< 20 μg/L). (**C** and **D**) The low Lamp2 subgroup had a significantly shorter RFS and OS than the high Lamp2 subgroup in the tissue microarray cohort with small hepatocellular carcinoma (SHCC, the diameter of HCC ≤ 5 cm).Statistical significance was assessed by two-sided log-rank tests.

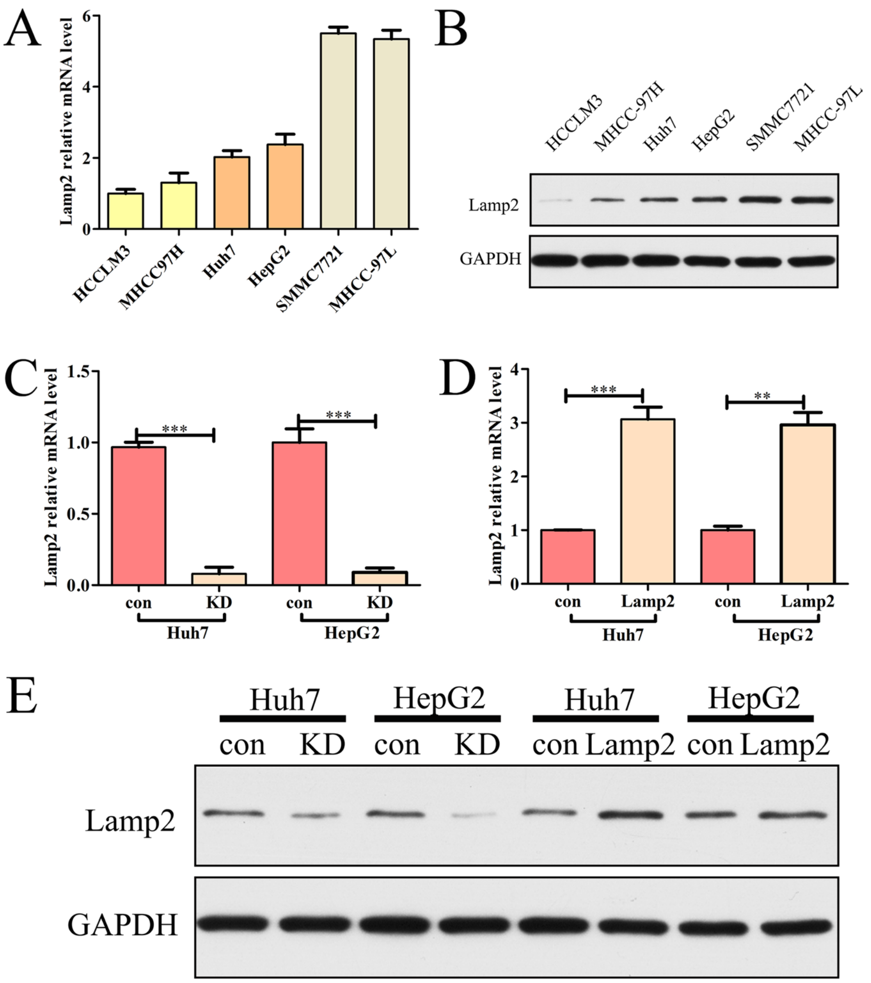

Fig-S2

Supplementary Figure 2: (**A**–**B**) qRT-PCR and Western blot analysis were performed to assess the expression of Lamp2 in several HCC cells. (**C**–**E**) qRT-PCR and Western blot analysis were performed to assess the knockdown and overexpression of Lamp2 in Huh7 and HepG2 cells.

Supplementary Table 1: Univariate analysis for RFS and OS

Variable	RFS			OS		
	Hazard Ratio	95% CI	*P* value	Hazard Ratio	95% CI	*P* value
**Age**, years, > 55 vs. ≤ 55	0.764	0.590–0.990	0.042	0.758	0.566–1.015	0.063
**Sex**, male vs. female	1.068	0.695–1.642	0.674	1.113	0.685–1.810	0.664
**HBsAg**, positive vs. negative	1.221	0.837–1.781	0.300	1.247	0.812–1.913	0.313
**Liver cirrhosis**, present vs. absent	1.127	0.867–1.467	0.371	1.401	1.036–1.895	0.029
**AFP**, µg/L	1.755	1.359–2.265	< 0.001	1.996	1.497–2.661	< 0.001
**Edmondson-Steiner classification**, III–IV vs. I–II	1.578	0.930–2.677	0.091	1.630	1.025–2.592	0.039
**Microvascular invasion**, present vs. absent	1.665	1.287–2.154	< 0.001	2.196	1.647–2.927	< 0.001
**Tumor diameter**, cm, > 5 vs. ≤ 5	2.540	1.937–3.331	< 0.001	1.573	1.174–2.109	0.002
**Tumor number**, multiple vs. solitary	3.122	2.365–4.121	< 0.001	2.451	1.816–3.307	< 0.001
**Capsule formation**, incomplete vs. complete	1.059	0.821–1.367	0.658	1.123	0.843–1.495	0.428
**Lamp2 expression**, low vs. high	1.831	1.417–2.366	< 0.001	2.181	1.632–2.916	< 0.001

Abbreviation: HBsAg, hepatitis B surface antigen; AFP, alpha fetoprotein.

Supplementary Table 2: Sequence of primers for real-time PCR

Gene Name	Forward primer (5′ -> 3′)	Reverse primer (5′ -> 3′)
**Lamp2**	GTGAGTTGTATTGGGGTTGATGTTA	CAATGATACTTGTCTGCTGGCTAC
**β-actin**	CTACCTCATGAAGATCCTCACCGA	TTCTCCTTAATGTCACGCACGATT
**E-cadherin**	CTTCCATGACAGACCCCTTAA	GAACGCATTGCCACATACACT
**ZO-1**	GGACTCGTATCTGTATGTGGG	AGAGCCTAATCTGACCTATGAAC
**N-cadherin**	GTCCTGGTCTTCTTCTCCTCC	CCTGCTTATCCTTGTGCTGAT
**Vimentin**	TCGTGATGCTGAGAAGTTTCG	TCTGGATTCACTCCCTCTGGT
**TGF-β**	AGAGCAACACGGGTTCAGGTA	TGGAAACCCACAACGAAATCT
